# Inflammatory myopathies in a patient with Darier disease, a possible association

**DOI:** 10.22088/cjim.9.2.201

**Published:** 2018

**Authors:** Kaveh Gharaei Nejad, Hojat Eftekhari, Rana Rafiei, Abbas Darjani, Narges Alizadeh

**Affiliations:** 1 Skin Research Center, Department of Dermatology, Razi Hospital, Guilan University of Medical Sciences, Rasht, Iran.

**Keywords:** Darier disease, Myositis, Dermatomyositis, Polymyositis

## Abstract

**Background:**

Darier disease (DD) is an autosomal dominant genetic disorder which develops from a mutation in the ATP2A2 gene. Inflammatory myopathies (IM) are the largest group of potentially treatable myopathies. In this case, we report development of IM in a patient with DD for the second time in the literature.

**Case presentation:**

The patient is a 59-year-old female, a known case of DD, who developed proximal muscle weakness 2 weeks prior to admission. Elevated muscle-enzymes, as well as typical electromyographic and radiologic confirmed the diagnosis of IM.

**Conclusions:**

Abnormalities in intracellular calcium homeostasis may explain the association between DM and DD, therefore it is noteworthy to keep this association in mind and conduct more research regarding this issue.

Darier disease (DD) is an autosomal dominant genetic disorder which develops from a mutation in the ATP2A2 gene causing a dysfunction in SERCA2 protein, interfering with cellular calcium signaling. It presents as brownish keratotic papules mainly on seborrheic areas of the face and trunk with onset during adolescence (1). The largest group of potentially treatable myopathies in children and adults is inflammatory myopathies (IM). They are classified based on the distinct clinicopathologic features, into four subtypes: dermatomyositis (DM), polymyositis (PM), necrotizing autoimmune myositis (NAM), and inclusion-body myositis (IBM) (2). Abnormalities in calcium homeostasis have been reported in patient with DM/PM (3) and IBM (4) and muscular dystrophies (5). Both diseases (DD and IM) have been associated with abnormalities in intracellular calcium homeostasis. Association of DD and IM has been reported once in the literature (4). To our knowledge, this is the second case of such occurrence. 

## Case presentation

**Figure 1:**
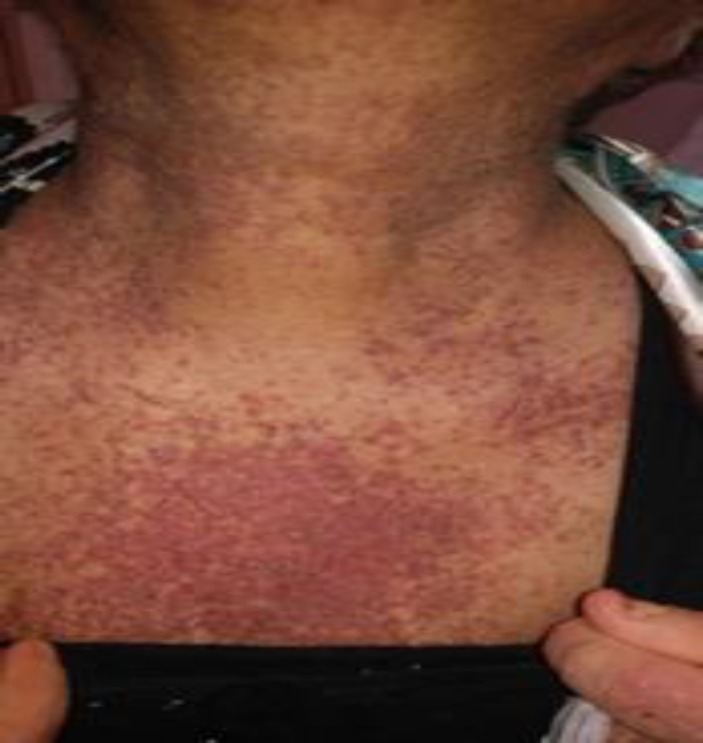
Keratotic papules over body, marker of Darier disease

The patient is a 59-year-old female, a known case of DD for 38 years, receiving occasional oral systemic retinoid (acitretin 25 mg twice weekly) for controlling DD-associated symptoms. She was relatively well until 2 weeks prior to admission when she developed fever, productive cough, worsening of skin lesions, generalized weakness and exertional dyspnea. On admission she was febrile (38.5°c), typical keratotic papules of DD were evident on face, scalp, infra-mammary and inguinal flexures and trunk. Some bullous and lichenoid papules were seen on the extensor aspects of distal extremities and erythematous patches over dorsal aspects of her fingers with sparing of the knuckles. Trachyonychia and V-shaped notches of the fingernails and toenails were prominent but no cuticular telangiectasia, capillary drop-outs or ragged borders were detected. She also had proximal-muscle weakness especially of pelvic muscles and a positive Gover's sign test (figure-1).

**Figure 2:**
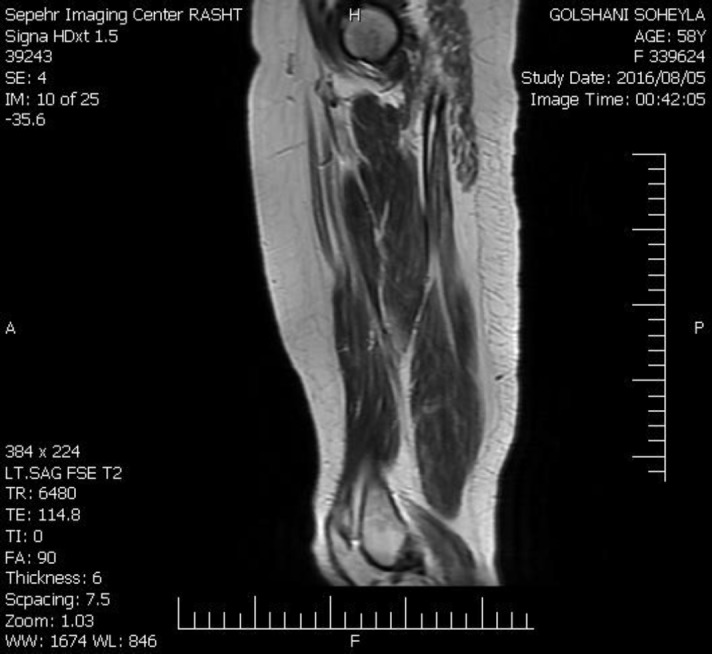
MRI of thigh. Signal alteration (low on T1) with feathery appearance of muscle fibers.

Initial laboratory evaluations revealed high serum titers of creatinine phosphokinase (5180 IU/L)), Lactate dehydrogenase (2099 IU/L) SGOT (281 IU/L), SGPT (162 IU/L), ESR (42mm/hr), aldolase (72 IU/L) levels. Antinuclear and antisynthetase, (JO-1, Mi-2) antibodies, were negative, but ,antibodies against signal recognition particle (SRP) and NXP2 (MJ-p 140-MU 140 kD protein) were positive. Electrodiagnostic investigation showed a myopathic process more prominent in proximal muscles without spontaneous activity. Serum markers and radiologic workups for occult malignancies were normal. In magnetic resonance imaging (MRI) of limbs, extensive and diffuse signal alteration (low on T1, high on T2) with feathery appearance accompanied by a little fluid accumulation between muscle fibers and intervening fascia of pelvic and shoulder girdle were seen (figure 2). 

**Figure 3:**
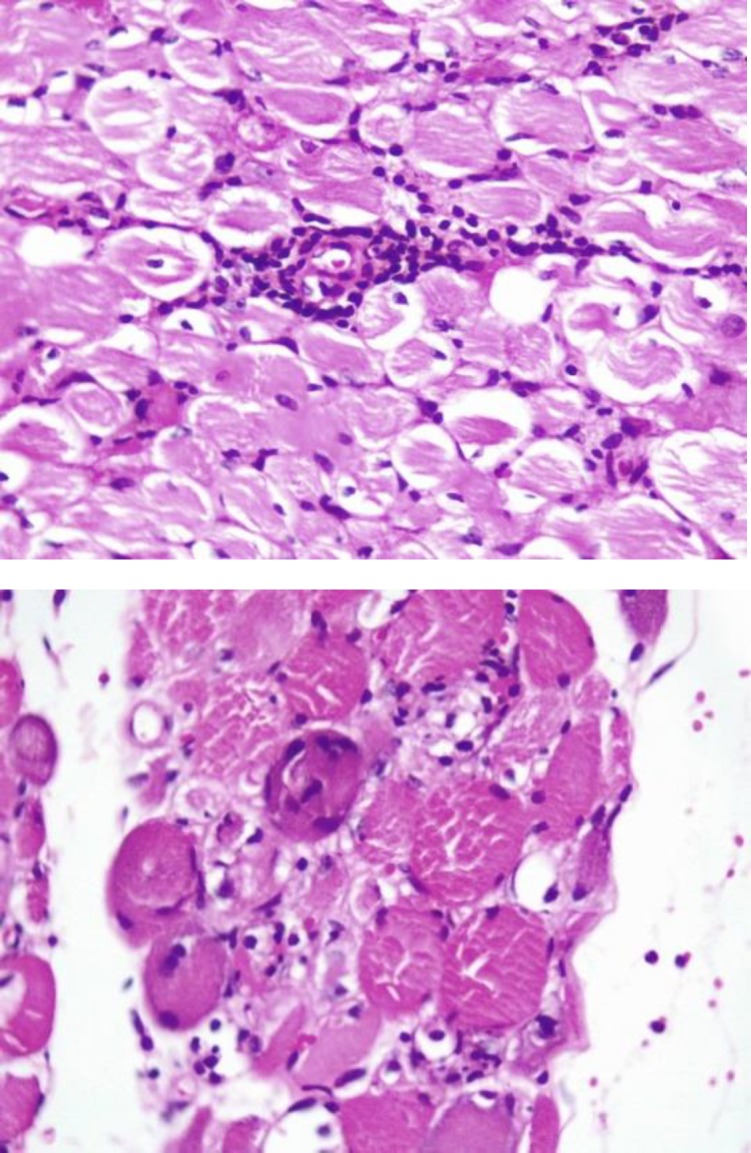
Biopsy of triceps. intrafascicular (right) and perivascular (left) T-cell infiltration and some necrotic and regenerating muscle fibers (H&E).

Biopsy of triceps confirmed the diagnosis of inflammatory myopathy with intrafascicular and perivascular T-cell infiltration and some necrotic and regenerating muscle fibers on hematoxylin and eosin (H&E) preparation (figure 3). 

She was admitted with the initial diagnosis of PM and received prednisolone (60 miligrams per day) and azathioprine (150 miligrams per day). Despite initial decrease in serum myositis-associated indices, patient's clinical status worsened and she developed dysphagia 3 weeks after admission, therefore, intravenous immunoglobulin (IVIG) and cyclophosphamide (50 miligrams per day) were added to her medications subsequently. Her condition improved gradually and she was discharged after 3 weeks. 

## Discussion

Darier disease (DD) develops from an autosomal dominant genetic mutation in the ATP2A2 gene, causing a dysfunction in SERCA2 protein. These inadequate SR/ER calcium stores disrupt cellular calcium signaling pathway, ultimately resulting in both acantholysis and apoptosis. The main clinical presentations of the disease are brownish keratotic papules mainly on seborrheic areas of the face and trunk (1). DD follows a prolonged course without spontaneous remission; however, severity may fluctuate with periods of improvement and worsening (7). 

DD is reported to be associated with neuropsychiatric disorders such as epilepsy and mood disorders and mutations in ATP2A2 gene have been shown in some families with psychiatric problems (8). SERCA2 haploinsufficiency and disorders in keratinocyte adhesion maybe related to rare malignant transformations in human papilloma virus –infected tissues in DD patients (9). Kalovidouris has shown calcium uptake by the sarco/endoplasmic reticulum (SER) in patients with DM is less than 50% than that of normal control subjects, resulting in high cytoplasmic calcium levels, similar to those observed in patients with DD. Abnormal cytoplasmic calcium concentrations in patients with PM/DM may be responsible for the cutaneous manifestations such as calcinosis cutis or may contribute to muscle weakness (3). 

In 2009, Lee et al. reported the development of DM in a known case of DD and proposed that abnormalities in calcium transport may explain the association between DM and DD (6). 

Intracellular calcium concentration is measured by optical (digital video microscopy, confocal laser scanning microscopy, and more recently multiphoton microscopy) and non-optical (electrophysiologic, calcium-selective electrodes, vibrating calcium-selective probe) procedures (10). 

We did not have the means to measure intracytoplasmic calcium concentration in our patient, therefore, whether this occurrence represents a true association or not, remains to be elucidated with further studies

In conclusion, both DD and IM have been linked to abnormalities of calcium homeostasis. We tried, as much as it was possible to document both pathologies by clinical and paraclinical evaluations available to our department, however, it was not possible to assess the intracellular calcium transfer pathways. We believe that it is noteworthy to conduct more research regarding this possible association and keep this association in mind. 

## References

[1] Savignac M, Edir A, Simon M, Hovnanianl A (2011). Darier disease: A disease model of impaired calcium homeostasis in the skin. Biochim Biophysi Acta.

[2] Dalakas MC (2015). Inflammatory muscle diseases. N Engl J Med.

[3] Kalovidouris AE (1984). Dysfunction of the sarcoplasmic reticulum in polymyositis. Arthritis Rheum.

[4] Amici DR, Pinal-Fernandez I, Mazala DG (2017). Calcium dysregulation, functional calpainopathy, and endoplasmic reticulum stress in sporadic inclusion body myositis. Acta Neuropathol Commun.

[5] Burr AR, Molkentin JD (2015). Genetic evidence in the mouse solidifies the calcium hypothesis of myofiber death in muscular dystrophy. Cell Death Differ.

[6] Lee WJ, Park GH, Lee MW (2009). Keratosis follicularis and dermatomyositis: Is there a common pathogenesis?. J Eur Acad Dermatol Venerol.

[7] Sehgal VN, Srivastava G (2004). Darier’s (Darier-White) disease/keratosis follicularis. Int J Dermatol.

[8] Jacobsen NJ, Lyons I, Hoogendoorn B (1999). ATP2A2 mutations in Darier's disease their relationship to neuropsychiatric phenotypes. Hum Mol Genent.

[9] Alexandrescu DT, Dasanu CA, Farzanmehr H, Kauffman CL (2008). Development of squamous cell carcinoma in Darier disease: a new model for skin carcinogenesis?. Br J Dermatol.

[10] Takahashi A, Camacho P, Lechleiter JD, Herman B (1999). Measurement of intracellular calcium. Physiol Rev.

